# Eggshell membrane for DNA sexing of the endangered Maleo ( *Macrocephalon maleo*)

**DOI:** 10.12688/f1000research.23712.4

**Published:** 2021-01-28

**Authors:** Pramana Yuda, Andie Wijaya Saputra

**Affiliations:** 1 Teknobiologi, Universitas Atma Jaya Yogyakarta, Kab. Sleman, DI Yogyakarta, 55281, Indonesia

**Keywords:** eggshell membrane, endangered birds, DNA sexing, Maleo

## Abstract

**Background:** Noninvasive DNA sampling has been applied across many avian genetic studies for a variety of purposes including conservation and management of endangered birds. However, its application in megapodes is still lacking. The previous genetic studies on megapodes used either blood or fresh tissue. Here we present the first demonstration of the use of eggshell membrane for research on endangered Maleo ( *Macrocephalon maleo*).

**Methods:** We used 24 post-hatched eggshell membranes collected from two different sites, Tambun and Tanjung Binerean, in North Sulawesi, 12 samples in each. Two different DNA extraction methods: alkaline lysis method and gSYNC ^TM^ DNA Extraction Kit **were applied.  To determine the sex of Maleo, we utilized PCR-based DNA sexing using CHD genes, with the primer set 2550F/2718R.

**Results:** We successfully extracted all samples; the mean sample concentration was 267.5 ng/µl (range 47–510.5 ng/µl) and samples were of high purity (A260/280 ratio 1.85±0.03). All samples were used to successfully identified sexes, 9 females and 15 males.

**Conclusions:** Our research clearly illustrates that eggshell membranes can be used for DNA sexing and open the possibility to build noninvasive DNA collections over large spatial scales for population study of endangered birds.

## Introduction

Studies on molecular ecology have a great impact on our knowledge on ecology and evolution of animals, i.e. the phylogenetic relationships and systematics of organisms, population genetics, mating systems, micro-evolutionary processes and host-parasite interactions ^1– 4^. Oftentimes a necessary prerequisite for answering evolutionary or ecological questions is access to a good DNA sample. Birds’ blood contains nucleated red blood cells with abundant DNA, making it a preferred source of DNA ^5^. However, obtaining blood requires the capture of a bird, which can provoke an increased level of stress and might results in unusual behavior or nest desertion. For example, blood sampling has been reported to reduce annual survival in Cliff Swallows ( *Petrochelidon pyrrhonota*) ^6^, although the effects of blood collection in free-living adult and immature birds is not thought to have major negative effects on adult survival, reproductive success, body condition, or behavior ^7^.

As an alternative to invasive sampling, researchers have adopted noninvasive sampling methods such as DNA capture from molted feathers ^8, 9^, feces ^10, 11^, and egg shell membrane ^12, 13^. In addition the application of moderately invasive sampling such as buccal swabs ^14, 15^ has also increased. Megapodes (family Megapodiidae) are a galliform clade, centered in Australasia ^16^, that are known for their unique super-precocial behavior ^17^. Megapodes are also renowned as the only bird species that use environmental heat rather than body temperature (brood) to incubate their eggs ^18^. Their ground-living habits, large body size and large egg size make them particularly vulnerable to human persecution, habitat destruction and habitat loss: 11 out of 21 species are now considered endangered or threatened in some form 19. Given this precarious conservation situation, the application of noninvasive DNA sampling techniques is crucial for megapode birds. Yet previous genetic studies on this family have used either blood ^20^ or fresh tissue ^21^.

Previously, all megapodes were assumed to be monogamous. The mating system is considered to correlate with sexual selection, with sexually dimorphic birds are non-monogamous and monomorphic birds are monogamous. The evolution of non-monogamous systems in birds was believed to be an adaptive solution to an unbalanced sex ratio ^22^. The sex-ratio in Maleo ( *Macrocephalon maleo*) is unknown, but based on previous assumptions, it is expected that the Maleo has an evenly balanced sex ratio. Even though Maleo are slightly sexual dimorphic, the available population data only report total population size and never mention sex ratio. A study on the correlation of incubation temperature and sex ratio of chicks has been carried out in the Australian brush-turkey ( *Alectura lathami*), which revealed that at average temperature the hatched chicks in the proportion of 1:1 of male and female chicks ^23^.

The purpose of our study was to determine whether the eggshell membrane of the endangered Maleo, a monotypic genus within the megapodes, could be successfully extracted and amplified for DNA sexing. Adult male and female Maleo are morphologically slightly different, but the chicks are not. To determine the sex in Maleo chicks, vent sexing has been conducted. Base on cloaca size and shape, a one-day-old male Maleo chick cloaca is bigger (3.96 ± 0.11 cm) and rounded, than the female cloaca (3.20 ± 0.10 cm), which is more oval in shape. The concentration of estrogen in female birds was also higher ^23^. Until recently, no molecular technique has been applied for sex determination of Maleo. As in all non-ratite birds, determination of sex in Maleo are based on heteromorphic Z and W chromosomes. Female birds are heterogametic sex ZW, meanwhile males are homogametic sex ZZ ^24, 25^. Maleo are endemic to Sulawesi, Indonesia ^16, 26^. The bird is a burrow-nesting megapode that incubates its eggs in communal nesting sites on beaches (coastal nesting grounds) and in soil heated by volcanic activity mostly at inland localities. Due to its small, severely fragmented population and continued rapid decline, the International Union for Conservation of Nature has classified Maleo as an endangered species ^27^. Among the major threats are the over-exploitation of eggs and loss of connectivity between forest and nesting grounds ^28^. To minimize these threats at some nesting grounds, conservation programs are currently removing eggs and hatching them in safer, semi-natural hatcheries, built close to the nesting grounds. These facilities provide an opportunity to collect non-invasive DNA samples from the eggshell membrane left in the soil or brought to the soil surface by the hatched Maleo. The DNA of the chicks can be obtained from the extraembryonic membranes that remain attached to eggshell membranes of post-hatched eggs. The extraembryonic membranes consist of the amnion, allantois and chorion, and form to support the life and growth of the bird embryo ^29^. During the embryonic development stage, the allantois sac expands, causing the inner shell membrane and chorion being combined and forming a chorioallantois membrane (CAM), which contains lots of blood capillaries ^29, 30^.

## Methods

### Study sites and genetic sampling

**Figure 1.**
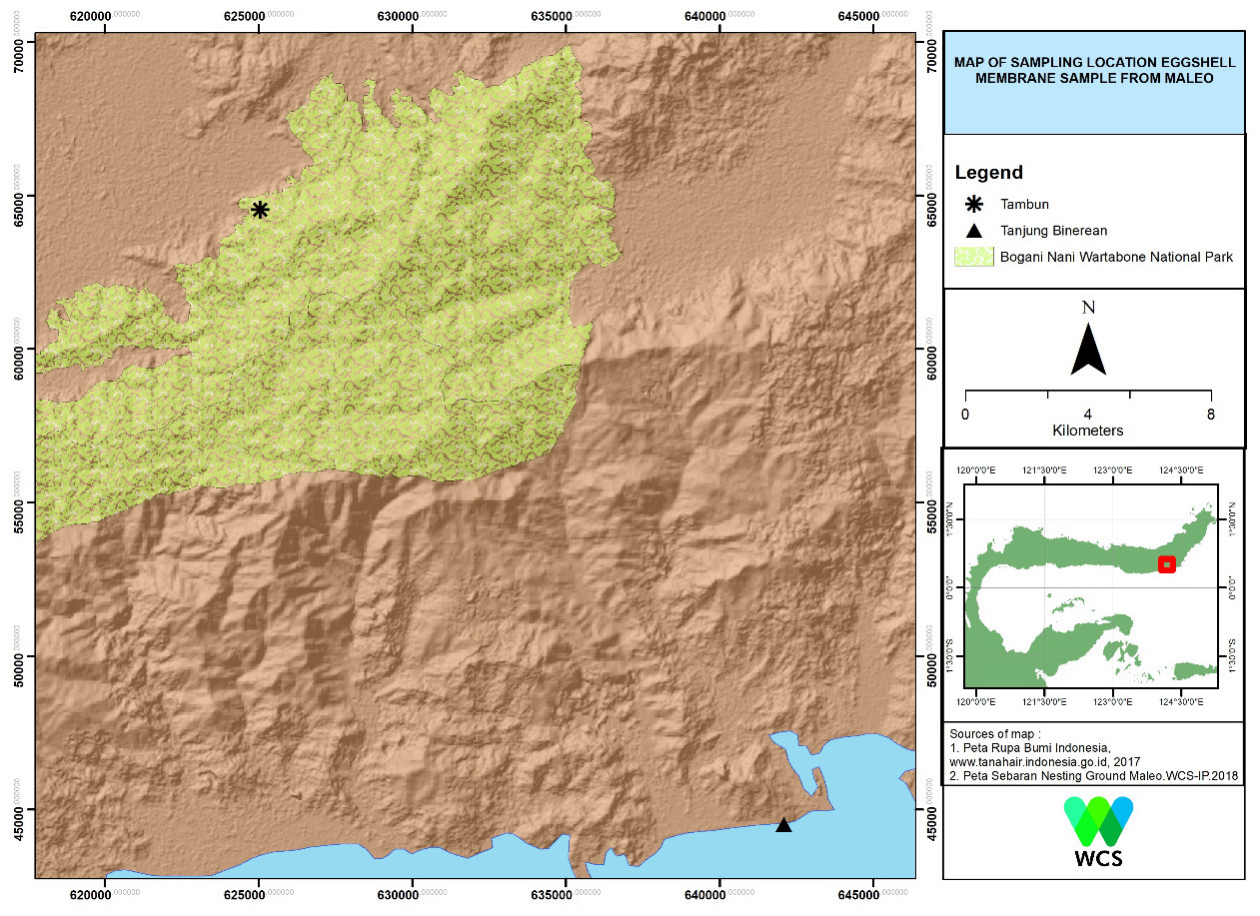
Sampling sites of eggshell membrane of Maleo ( *Macrochepalon maleo*) in North Sulawesi, Indonesia.

Post-hatched egg-shell membranes were collected from semi-natural hatcheries of Maleo at two different nesting grounds: an inland geothermal heated nesting ground at Tambun (Bogani Nani Wartabone National Parks and a sun-heated sand beach nesting ground at Tanjung Binerean, North Sulawesi, Indonesia ( Figure 1). All samples were collected from 4 ^th^ April until 1 ^st^ May 2018. Samples were egg-shell membranes from maleo chicks that hatched less than 24 hours earlier based on records of staff from semi-natural hatcheries. The samples were collected from the surface and inside the substrate (soil and sand) where the eggs are laid. To prevent post-sampling contamination, each sample was placed separately in a zip-lock plastic bag and stored in silica gel for delivery to laboratory. The samples were stored at -40°C until DNA extraction were conducted.

### DNA extraction

We used two different DNA extraction methods: the alkaline lysis method and gSYNC™ DNA Extraction Kit (Genaid). For the alkaline lysis method, we followed the recommended procedure for rapid preparation of mouse tails or nail lysates suitable for amplification using DNA polymerase from hyperthermophilic archaeon *Pyrococcus kodakaraensis* (KOD FX Neo 1103; TOYOBO Co. Ltd.). The eggshell membrane used was consisted of dry chorioallantois membrane (CAM), included allantois blood vessels, as DNA materials of the chicks, except five samples from Binerean (MB04, MB09, MB10, MB11 and MB12) with no blood vessels. The membrane (20–25 mg) was grinded using a micro-pestle in a 1.5 mL microcentrifuge tube; next, 180 µL NaOH (50 mM) was added, the suspension mixed thoroughly by vortexing and then incubated at 90°C in water-bath for 10–29 min. Following this, 20 µL Tris-HCl (1 M, pH 8.0) was added and the tube was vortexed thoroughly, then centrifuged at 12,000 RPM for 5 min. Lysate was removed to new 1.5 mL microtube and store at freezer until used for PCR.

Meanwhile the protocol for gSYNC™ DNA Extraction Kit (Genaid) followed the provided user manual with little modifications. The eggshell membrane (25 mg) was grinded using a micro-pestle in a 1.5 mL microcentrifuge tube; 300 µl GST Buffer (Tris, SDS) and 30 µl Proteinase K (10 mg/ml) was added to the sample mixture, mixed thoroughly by vortexing and incubated at 60°C in water-bath overnight or until the tissue was lysed completely. Next, 200 µl GSB Buffer was added to the sample mixture, mixed thoroughly by pulse-vortexing and incubated at 70°C for 10 min. After this, 200 µl ethanol (100%) was added to the sample mixture, which was mixed thoroughly by pulse-vortexing and brief spinning of the tube to remove drops from the inside of the lid. Next, a GS Column was placed in a Collection Tube and the mixture (including any precipitate) was carefully transferred to the GS Column, which was centrifuged at 14,000 RPM for 1 min then the GS Column was placed in a new Collection Tube. Following this, 400 µl W1 Buffer was added to the GS Column and centrifuged at 14,000 RPM for 1 min then flow-through was discarded. Next, 750 µl Wash Buffer was added to the GS Column, centrifuged at full speed for 1 min, then the flow-through was discarded, the tube centrifuged at 14,000 RPM for an additional 3 min to dry the column, 50 µl of preheated Elution Buffer (pH 7.5–9.0) added to the membrane of the GS Column. The GS Column was then left to stand for 3 min, following a final centrifugation at full speed for 2 min to elute the DNA.

The eluded DNA (1 µl) was quantified using NanoVue Plus™ (Biochrom, Harvard Bioscience, Inc), at A260 nm. The 260/280 nm absorbance ratio was also measured to give an indication of purity of the DNA. Pure DNA has expected ratios of 1.7–1.9.

### DNA sexing

To determine the sex of Maleo, we applied PCR based DNA sexing by using CHD genes, with the primer set 2550F/2718R ^31^. PCR used a 10 µl total volume containing 1 µl (30 ng/µl) diluted template DNA (genomic DNA or lysate), 1.2 µl sterile dH _2_O, 5 µl 2x PCR buffer KOD FX Neo, 2 µl dNTPs (2 mM), (TOYOBO Co. Ltd.), 0.3 µl Primer 2550F (10 µM; 5'-GTT ACT GAT TCG TCT ACG AGA-3'), and 0.3 µl Primer 2718R (10 µM; 5'-ATT GAA ATG ATC CAG TGC TTG-3', ^31^), and 0.2 U KOD polymerase enzyme. PCR was carried out in a Veriti™ 96-well thermal cycler (Applied Biosystems™). For genomic DNA templates, the following profile was used: 1 cycle at 94°C for 2 min followed by 35 cycles of 98°C for 10 sec, 53°C for 30 sec and 68°C for 45 sec;, and a final extension at 68°C for 7 minutes. For lysate as DNA template, the PCR profiles was the same for DNA genome, except that it was run for more cycles (40x). We employed egg-shell membranes from the domestic chicken as a positive control. The positive control consistently identified as a female with the same PCR condition in this research.

Amplification of CHD Genes were resolved on a 2% agarose gel. Electrophoresis was conducted using TAE (0.5×) buffer, stained by ethidium bromide (1%), at 100 V for 30 minutes; and 5 µl PCR product was mixed with 1 µl loading dye *.* After finish, the gel was visualized and analyzed on Gel Logic 200 Imaging System and Kodak Molecular Imaging Software. To confirm that the amplified fragments were the CHD genes, the PCR products of one male and one female sample, respectively, were sequenced. The gels were cut on upper and lower bands for female samples and the single band for male sample, then purified for sequencing. The sequence reactions were carried for both direction in sequencing services laboratory provided by 1 ^st^ BASE Laboratories (Apical Scientific Sdn Bhd, Malaysia). The sequences were check and edited manually on Bioedit version 7.0.5.3 ^32^ and Chromas versi 2.6.5 (Technelysium Pty Ltd). Sequence similarity was probed using NCBI BLAST ^33^.

## Results

All eggshell membranes were successfully extracted, with mean DNA concentrations around 267.5 ng/µl (range 47–510.5 ng/µl). The average DNA concentration extracted from eggshell membrane collected from coastal nesting grounds (Tanjung Binerean: 213±179 ng/µl,) was significantly lesser than of that of inland nesting grounds (Tambun: 322±153 ng/µl, p=0.004; Data Supp.1). These results demonstrate that all samples were adequate for further PCR based analysis.

**Figure 2.**
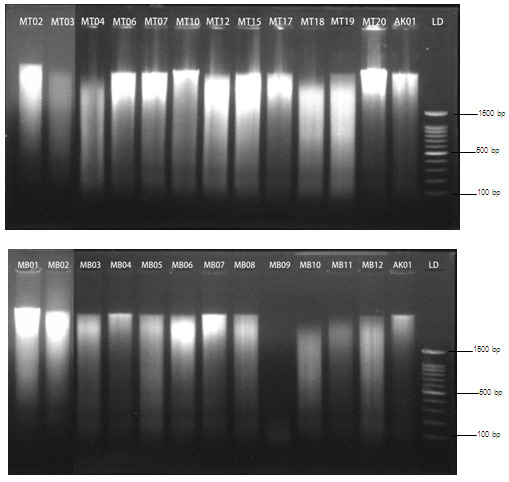
Gel visualizing DNA degeneration of eggshell membrane samples. Above: Tambun; Below: Tanjung Binereaan; LD: 100 bp DNA Ladder (SMOBiO); AK01: domestic chicken eggshell membrane.

The 260/280 nm absorbance ratio of all samples ranged from 1.81 to 1.89, with an average of 1.85 (±0.03). Meanwhile the average for Tambun and Tanjung Binerean samples were, respectively, 1.85 (±0.03) and 1.84 (±0.01); Table 1). This result suggested good purity of DNA extracted from eggshell samples. However. gel visualization of extracted DNA showed smears in all samples ( Figure 2), pointing to some DNA degradation.

**Table 1.** Concentrations and purity of DNA extracted from eggshell membrane of Maleo ( *Macrochepalon maleo*).

No.	Location	Sample Code	Purity (A260/A280 Ratio)	Concentration (ng/µl)
1	Tambun	MT03	1.848	127.5
2	Tambun	MT02	1.821	204.0
3	Tambun	MT17	1.879	280.0
4	Tambun	MT18	1.845	302.5
5	Tambun	MT10	1.822	308.0
6	Tambun	MT04	1.881	308.5
7	Tambun	MT12	1.829	342.0
8	Tambun	MT06	1.869	349.5
9	Tambun	MT07	1.898	364.5
10	Tambun	MT19	1.832	381.0
11	Tambun	MT20	1.846	383.0
12	Tambun	MT15	1.887	510.5
Average (Tambun)			1.855	321.75
13	Tanjung Binerean	MB09	1.880	47.0
14	Tanjung Binerean	MB11	1.859	72.5
15	Tanjung Binerean	MB10	1.813	155.0
16	Tanjung Binerean	MB08	1.818	174.4
17	Tanjung Binerean	MB04	1.839	183.0
18	Tanjung Binerean	MB07	1.844	206.5
19	Tanjung Binerean	MB12	1.858	209.0
20	Tanjung Binerean	MB05	1.841	232.0
21	Tanjung Binerean	MB02	1.821	249.5
22	Tanjung Binerean	MB03	1.852	250.0
23	Tanjung Binerean	MB06	1.816	286.0
24	Tanjung Binerean	MB01	1.893	495.0
Average (Tanjung Binerean)			1.845	213.3
**Average (all samples)**			**1.850**	**267,5**

### Sex determination

**Figure 3.**
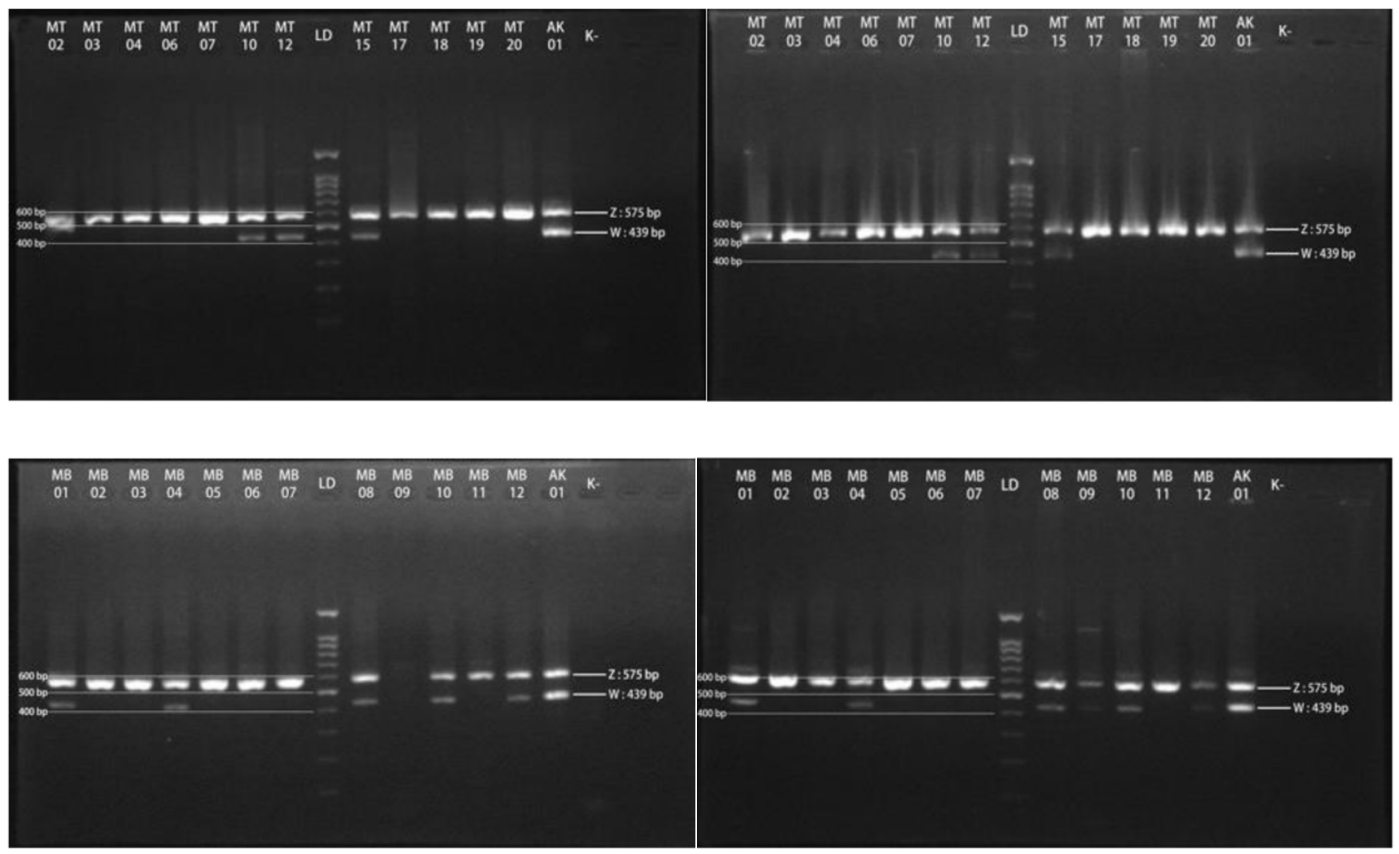
Gel visualizing of CHD-genes amplification of Maleo using extracted DNA (left) and Lysate (right) for molecular sexing of Tambun (above) and Tanjung Binerean (below) samples. AK1, positive control, female chicken; K, negative control, no template.

Out of 24 samples in which extracted DNA was used as a template, one sample (MB09) was not amplified. Meanwhile all samples based on lysate were successfully amplified. There was complete agreement in gender determination across all Maleo samples that were run with different DNA template ( Figure 3). Females showed two bands (545 bp and 395 bp), whereas males exhibited one band (545 bp). Three sequences of CHD1 genes have been deposited in GenBank (accession numbers MT074328, MT074329 and MT074330). Sequence similarity searches on the upper band revealed a match with CHD-Z genes of other bird species (i.e. *Anser cygnoides*, *Anser reevesii, Anas penelope*). Meanwhile the lower band matched CHD-W submissions of other birds (i.e. *Gallus gallus, Crossoptilon mantchuricum*, *Syrmaticus reevesii*).

In total 9 samples were identified as females and 15 were males ( Table 2). Four out of five samples with no allantois blood vessels were identified as females. Based on this limited sample, the sex ration of Maleo’s chicks in Tambun and Tanjung Binerean is biased towards males. The sex ratio males to females was 1.6. Care should be taken using eggs to identify the sex of the chicks. The use of freshly laid eggs resulted in inaccurate estimates of the primary ratio, due to contaminants from the DNA of the hen ^34^. In the other hand, using DNA isolated from CAM were reliable to sex the newly hatched Denizli chicken. The results of DNA sexing of chicks in all samples matched with that determined by morphological appearance of gonads ^35^.

**Table 2.** Identified sex of Maleo ( *Macrocepalon maleo*) based on molecular sexing with different templates.

No.	Sample Code	Template	
		Extracted DNA	Lysate
1	MT02	Male	Male
2	MT03	Male	Male
3	MT04	Male	Male
4	MT06	Male	Male
5	MT07	Male	Male
6	MT10	Female	Female
7	MT12	Female	Female
8	MT15	Female	Female
9	MT17	Male	Male
10	MT18	Male	Male
11	MT19	Male	Male
12	MT20	Male	Male
13	MB01	Female	Female
14	MB02	Male	Male
15	MB03	Male	Male
16	MB04	Female	Female
17	MB05	Male	Male
18	MB06	Male	Male
19	MB07	Male	Male
20	MB08	Female	Female
21	MB09	-	Female
22	MB10	Female	Female
23	MB11	Male	Male
24	MB12	Female	Female

## Discussion

This study has demonstrated the first successful DNA isolation from eggshell membranes of a megapode bird. Our success rate (100%) compares favorably to that of previous avian eggshell membrane studies of Black-tailed Godwits ( *Limosa limosa*), which also successfully extracted DNA from all 47 eggshell membranes ^13^. ****The freshness of the samples might be one of the determinant factors of DNA extraction success. Our samples were relatively fresh, extracted 5–15 days after collection and kept in the freezers until extraction, with no concomitant extraction failure and high purity (1.85±0.03) and concentration (267.5 ng/µl) of DNA.

PCR amplification of CHD genes succeeded in 96% of the eggshell membrane samples, with only one eggshell membrane isolate out of 24 failing to amplify. The quantity of the DNA sample (MB09; 47 ng/µl) might be the cause of the failure, since its quality was relatively good (1.88). However, using lysate as DNA template for PCR resulted in 100% amplifications across 24 samples. This result show that eggshell membrane isolates yielded DNA with little amplification problems. Compared to blood DNA isolates, eggshell membrane DNA isolates of Black-tailed Godwits ( *Limosa limosa*) also yielded fewer amplification problems ^13^. One eggshell membrane DNA isolate out of 21 and 3 samples of Black-tailed Godwit ( *Limosa limosa*) did not amplify for 2 and 5 of the 11 microsatellite loci. The amplification success rates was 99.1% ^13^. Meanwhile the success rate of eggshell membrane of Sage Grouse ( *Centrocercus uropihasianus*) for DNA sexing was only 55.6% ^12^.

This study demonstrates that hatched eggshell membrane provides useful noninvasive DNA material as an alternative to invasive sampling in sex determination studies of Maleo. Information of the sex of the hatched eggs are important to understand demographic issues, such as the demographic consequences of offspring sex ratio bias or whether there is any sex-specific mortality or dispersal. Furthermore, this information is very important for translocation programs of endangered species, including Maleo.

This study provides additional evidence that noninvasive DNA samples yield reliable results and eliminating the need for capture and invasive sampling. Collection of post-hatched eggshell membrane of Maleo, and other megapodes does not require specific skills. This noninvasive DNA sampling also open the possibility to build participation of local community or local conservation area staff on DNA collections over large spatial scales. Furthermore, the collected samples provide sufficient samples required for population and other ecological and evolutionary study of endangered bird species.

## Data availability

### Underlying data

NCBI GenBank: Macrocephalon maleo isolate MT10-FTB_UAJY_01 chromo-helicase DNA binding protein (CHDZ) gene, partial cds. Accession number MT074328.

NCBI GenBank: Macrocephalon maleo isolate MB01-FTB_UAJY_02 chromo-helicase DNA binding protein (CHDZ) gene, partial cds. Accession number MT074329.

NCBI GenBank: Macrocephalon maleo isolate MT10-FTB_UAJY_03 chromo-helicase DNA binding protein (CHDZ) gene, partial cds. Accession number MT074330.

Mendeley Data: Eggshell membrane for DNA sexing of the endangered Maleo (Macrocephalon maleo). http://doi.org/10.17632/mjp5n9pcj3.

This project contains the following underlying data:

- Electrophoresis photos. (Folder containing photos of DNA extraction and PCR sexing gels.)
- Sequences. (Folder containing raw sequencing files.)

Data hosted with Mendeley Data are available under the terms of the Creative Commons Attribution 4.0 International license (CC-BY 4.0).
