# Supplementary material for: Eggshell membrane for DNA sexing of the endangered Maleo ( Macrocephalon maleo)
Source: F1000Res. 2021 Jan 28;9:599. Originally published 2020 Jun 12. [Version 4] doi: 10.12688/f1000research.23712.4 (PMC7836084; doi:10.12688/f1000research.23712.4)
Supplement: Peer review history

# Peer review: Reviewer response for version 3

**DOI:** 10.5256/f1000research.31413.r76671

**Published:** 2021-01-21

**Authors:** Jessica Worthington Wilmer

**Affiliations:** 1 Biodiversity and Geosciences Program, Queensland Museum, South Brisbane, Qld, Australia

This revision has incorporated many of the suggestions from my previous review and I now approve the manuscript.

 I just have a few very minor language/writing suggestions that should be made to improve the English and information provided.

**Introduction:**

1. "Megapodes are also known as the only bird species that are not incubating their eggs, but make use environmental heat to incubate their eggs".
  Please rewrite this sentence as follows:
  "Megapodes are also renowned as the only bird species that use environmental heat rather than body temperature (brood) to incubate their eggs".
2. "The DNA of the chicks can be obtained from the extraembryonic membranes of fertilized egg, which was attached on the hatched eggshell membrane. The extraembryonic membranes were consisted of amnion, allantois and chorion, and formed to support the life and growth of the bird embryo". 
  Please rewrite these 2 sentences as follows:
  ""The DNA of the chicks can be obtained from the extraembryonic membranes that remain attached to eggshell membranes of post-hatched eggs. The extraembryonic membranes consist of the amnion, allantois and chorion, and form to support the life and growth of the bird embryo".

Is the work clearly and accurately presented and does it cite the current literature?

Partly

If applicable, is the statistical analysis and its interpretation appropriate?

Not applicable

Are all the source data underlying the results available to ensure full reproducibility?

No source data required

Is the study design appropriate and is the work technically sound?

Partly

Are the conclusions drawn adequately supported by the results?

Yes

Are sufficient details of methods and analysis provided to allow replication by others?

Yes

Reviewer Expertise:

Population genetics, molecular systematics, landscape genetics, evolutionary biology, conservation biology, DNA sexing (megapodes).

I confirm that I have read this submission and believe that I have an appropriate level of expertise to confirm that it is of an acceptable scientific standard.

# Peer review: Reviewer response for version 2

**DOI:** 10.5256/f1000research.29940.r74177

**Published:** 2020-11-18

**Authors:** Jessica Worthington Wilmer

**Affiliations:** 1 Biodiversity and Geosciences Program, Queensland Museum, South Brisbane, Qld, Australia

This paper examines the capacity to genetically sex Maleo ( *Macrocephalon maleo*) chicks from DNA extracted from the membranes of hatched eggs. This is interesting work and potentially very important for understanding hatching sex ratios of Maleo and for future conservation work of this endangered megapode.

 The paper has already gone through one round of review and is generally well presented, however, there are a couple of fundamental issues that need to be highlighted and then addressed by the authors.

 1) Egg-Chick membrane anatomy and biology:

- It states in the title and throughout the paper that eggshell membranes are being used but the authors must be careful in implying that the eggshell membranes themselves are the source of chick DNA. The shell membranes are produced by the hen when the shell is formed in the oviduct and, therefore, will always be female. Thus, eggshell membranes on their own without the inclusion embryonic tissue will not be useful for DNA sexing of hatched chicks. So, the source of the chick DNA extracted must be from one of the extraembryonic membranes or some other chick tissue left behind in the shell at hatching. This fundamental egg-biology information must be included in the introduction.
- The issue becomes clearer in the methods section where the authors detail the tissue used for DNA extraction “The eggshell membrane (20-25mg; *mostly with dry allantois blood vessel* ) was grinded...” This is the critical information as these blood vessels comprise the extraembryonic arterial system and therefore are the source of chick DNA in this study. The question then is did the authors specifically target these sections of the eggshell membranes because of the blood vessels (with reference to the fundamental egg-chick biology)? If so, then this should be clearly stated as what needs to be done when dealing with hatched eggs.
- “Mostly” is not an accurate scientific term. Please provide detail as to how many eggshell membranes included allantois blood vessels out of the total number (24) of samples. This is important because of the 9 females that were detected. How many of the samples that didn’t include allantois blood vessels were sexed as being female? Those membranes could still include other extraembryonic tissues however, there needs to be a way for the authors to demonstrate with confidence that it is the chick that was sexed as female and not because they have amplified the sex chromosomes of the hen. (see Arnold et al, 2003 ^1^).

2) Maleo and Megapodes

- The introduction needs to include the fundamental biological information that megapodes are the only avian species/group that are not brood incubators (Frith 1956 ^2^). All megapodes employ environmental heat to incubate their eggs. Temperature dependent sex biased mortality has been shown to occur in the Australian Brush Turkey (Eiby et al. 2008 ^3^) and it may well be that further in depth studies of Maleo may reveal a similar mechanistic interaction between their environmental incubation temperature and hatching sex ratios. The current observed skew in hatching ratio is interesting although the low sample numbers may mean it is not biologically / statistically significant.
- It would also be good to include some basic information regarding sex determination in birds and that in megapodes, as in all other non-ratite birds, sex is determined by heteromorphic Z and W chromosomes (Belter and Deboer, 1984 ^4^) and, unlike in mammals, females are the heterogametic sex ZW and Males ZZ.

Is the work clearly and accurately presented and does it cite the current literature?

Partly

If applicable, is the statistical analysis and its interpretation appropriate?

Not applicable

Are all the source data underlying the results available to ensure full reproducibility?

No source data required

Is the study design appropriate and is the work technically sound?

Partly

Are the conclusions drawn adequately supported by the results?

Yes

Are sufficient details of methods and analysis provided to allow replication by others?

Yes

Reviewer Expertise:

Population genetics, molecular systematics, landscape genetics, evolutionary biology, conservation biology, DNA sexing (megapodes).

I confirm that I have read this submission and believe that I have an appropriate level of expertise to confirm that it is of an acceptable scientific standard, however I have significant reservations, as outlined above.

## References

[1] : Primary sex ratios in birds: problems with molecular sex identification of undeveloped eggs. *Mol Ecol*.2003;12(12) : 10.1046/j.1365-294x.2003.02007.x 3451-8 10.1046/j.1365-294x.2003.02007.x 14629359

[2] : BREEDING HABITS IN THE FAMILY MEGAPODIIDAE. *Ibis*.2008;98(4) : 10.1111/j.1474-919X.1956.tb01453.x 620-640 10.1111/j.1474-919X.1956.tb01453.x

[3] : Temperature-dependent sex-biased embryo mortality in a bird. *Proc Biol Sci*.2008;275(1652) : 10.1098/rspb.2008.0954 2703-6 10.1098/rspb.2008.0954 18755669PMC2605828

[4] : A karyological study of 55 species of birds, including karyotypes of 39 species new to cytology.1984;

# Peer review: Reviewer response for version 2

**DOI:** 10.5256/f1000research.29940.r72362

**Published:** 2020-10-20

**Authors:** Sena Adi Subrata

**Affiliations:** 1 Department of Forest Resource Conservation, Faculty of Forestry, Universitas Gadjah Mada, Yogyakarta, Indonesia

The authors have provided substantial correction. I agree with all revisions. I suggest a minor improvement in Figure 3 : characters representing length of ladder should be clear enough. At present, it is hard to read the characters.

Is the work clearly and accurately presented and does it cite the current literature?

Yes

If applicable, is the statistical analysis and its interpretation appropriate?

No

Are all the source data underlying the results available to ensure full reproducibility?

Partly

Is the study design appropriate and is the work technically sound?

Partly

Are the conclusions drawn adequately supported by the results?

Partly

Are sufficient details of methods and analysis provided to allow replication by others?

Partly

Reviewer Expertise:

Wildlife molecular ecologist

I confirm that I have read this submission and believe that I have an appropriate level of expertise to confirm that it is of an acceptable scientific standard.

# Peer review: Reviewer response for version 1

**DOI:** 10.5256/f1000research.26162.r69914

**Published:** 2020-09-08

**Authors:** Sena Adi Subrata

**Affiliations:** 1 Department of Forest Resource Conservation, Faculty of Forestry, Universitas Gadjah Mada, Yogyakarta, Indonesia

In general, the article provided a new finding that never been reported, and discussed possible contribution of the finding to the species conservation. However, there was unclear information particularly in the methods section that should be resolved before the article can be published.

 Here is my suggestion:

- In the introduction section, a correction is needed on the family name of the bird.
- In methods section, particularly genetic sampling, a further explanation is required regarding sample collection: how to collect the membranes ? How old is the samples ?  Furthermore, the author should refer supernatant as lysate. Volume and DNA concentration should be explicitly mentioned when describing PCR condition. Method of sex determination of positive control (egg shell membrane of chicken) should be described. Report of statistical test should be removed because it was meaningless without argument in the discussion.
- The author should mention and analyze a failed sample in the result and discussion sections. Most likely it correlated with DNA quality and quantity. When comparing sex ratio, it should be done in the same way.
- In the discussion section, particularly when discussion freshness of a sample, the author should provide data on the old of the sample. In the Figure 3, a scale indicating length of the amplicon/ladder should is required. 

Is the work clearly and accurately presented and does it cite the current literature?

Yes

If applicable, is the statistical analysis and its interpretation appropriate?

No

Are all the source data underlying the results available to ensure full reproducibility?

Partly

Is the study design appropriate and is the work technically sound?

Partly

Are the conclusions drawn adequately supported by the results?

Partly

Are sufficient details of methods and analysis provided to allow replication by others?

Partly

Reviewer Expertise:

Wildlife molecular ecologist

I confirm that I have read this submission and believe that I have an appropriate level of expertise to confirm that it is of an acceptable scientific standard, however I have significant reservations, as outlined above.

# Response

**Published:** 2020-09-28

**Authors:** Pramana Yuda

**Affiliations:** Universitas Atma Jaya Yogyakarta, Indonesia

Thank you for reviewing our article and some valuable recommendations for improvement.  We have incorporated the correction and recommendations in our revised article. 

- Family name was corrected
- In method section we added description of sample age, collection technique, the volume and concentration of the DNA, and the more description on positive control for sex determination.    We also removed the statistical test.
- Failed sample (MB09) in sex determination was added in ‘Sex determination’ section and discussion.    
- Length of amplicon was added in Figure 3
- The description on age and freshness of samples were added in method and discussion sections.
